# Glucocorticoid receptor polymorphisms modulate cardiometabolic risk factors in patients in long-term remission of Cushing’s syndrome

**DOI:** 10.1007/s12020-016-0883-z

**Published:** 2016-02-13

**Authors:** Sean H. P. P. Roerink, M. A. E. M. Wagenmakers, J. W. A. Smit, E. F. C. van Rossum, R. T. Netea-Maier, T. S. Plantinga, A. R. M. M. Hermus

**Affiliations:** Division of Endocrinology, Department of Medicine, Radboud University Medical Centre, Geert Grooteplein 8, PO Box 9101, 6500 HB Nijmegen, The Netherlands; Division of Endocrinology, Department of Internal Medicine, Erasmus University Medical Center Rotterdam, ‘s-Gravendijkwal 230, 3015 CE Rotterdam, The Netherlands

**Keywords:** Cushing’s syndrome, Long-term remission, Glucocorticoid receptor polymorphisms, Genetic predisposition, Metabolic profile

## Abstract

**Context:**

Glucocorticoid receptor (GR) polymorphisms modulate glucocorticoid (GC) sensitivity and are associated with altered metabolic profiles.

**Objective:**

To evaluate the presence of GR polymorphisms (*Bcl*I (rs41423247), N363S (rs56149945), ER22/23EK (rs6189/rs6190), and 9*β* (rs6198) and investigate their associations with metabolic alterations in patients in long-term remission of Cushing’s syndrome (CS).

**Design and setting:**

Cross-sectional case–control study.

**Patients and methods:**

Sixty patients in long-term remission of CS were genotyped. Associations between GR polymorphisms and multiple vascular, body composition and metabolic parameters were investigated.

**Main outcome measures:**

Allelic frequencies of the polymorphisms and their associations with several cardiometabolic risk factors.

**Results:**

This study shows that carriers of the 9*β* polymorphism have a higher systolic blood pressure and lower resistin levels. The GC sensitizing *Bcl*I polymorphism is associated with an adverse cardiometabolic risk factor profile: higher fat percentages of extremities and legs, higher serum leptin and E-selectin levels, and higher intima media thickness in carriers versus non-carriers.

**Conclusions:**

The 9*β* and *Bcl*I polymorphisms of the GR adversely affect the cardiometabolic profile in patients who are in remission after the treatment of CS. This suggests that genetically altered GC sensitivity modulates the long-term adverse cardiometabolic effects resulting from (endogenous) hypercortisolism.

## Introduction

Cushing’s syndrome (CS) is a disorder resulting from chronic exposure to increased levels of glucocorticoids (GC), frequently caused by an ACTH-producing pituitary adenoma [Cushing’s disease (CD)] or by primary adrenal overproduction of cortisol [adrenal CS (ACS)] [1]. CS is associated with body composition changes, cardiometabolic abnormalities such as type 2 diabetes mellitus (T2DM), hypertension and dyslipidemia, and ultimately cardiovascular disease [2]. We [3] and others [4, 5] have shown that many adverse metabolic and cardiovascular characteristics, and body compositional changes persist after treatment, even after long-term remission. Although these adverse metabolic and cardiovascular characteristics are common in these patients, their incidence and severity vary among patients. This variation seems not to be explained by differences in cortisol excess or disease duration alone. Therefore, a variable sensitivity to GC possibly plays a role in modulating the effect of cortisol excess [6].

Several investigations in *healthy* subjects have shown that GR polymorphisms are associated with altered GC sensitivity and alterations in metabolic profiles and body composition. The *Bcl*I and N363S polymorphisms of the GR gene have been associated with enhanced sensitivity to GC, increased abdominal obesity, an adverse lipid profile, and hyperinsulinemia [7–10]. In contrast, the ER22/23EK GR polymorphism has been associated with GC resistance and a favorable metabolic profile and body composition [11, 12]. The 9*β* polymorphism is associated with increased expression and stabilization of the dominant-negative splice variant GR-β. Enhanced GR-β expression results in greater inhibition of GR-α transcriptional activity, and as GR-α is the functional GR isoform, in relative GC resistance [13–15]. This polymorphism is associated with increased serum levels of inflammatory parameters and cardiovascular disease despite a more favorable lipid profile in men and body composition in women [16, 17].

The functional role of GR polymorphisms has been extensively studied in the general healthy population. In contrast, only a small number of studies have been performed on the functional role of GR polymorphisms in active CS and CS in remission. These few studies have found an association of the 9*β* polymorphism with the risk of developing diabetes mellitus [18] and of the *Bcl*I polymorphism with increased skeletal GC sensitivity and worse cognitive performance [19, 20].

We hypothesize that differences between patients in the severity of the adverse metabolic and vascular profile after cure of CS are related to differences in GC sensitivity due to GC receptor polymorphisms. Therefore, we investigated the associations of these genetic variants with the presence/persistence of the adverse metabolic and vascular profile and body composition after long-term remission of CS.

## Subjects and methods

Sixty adult (>18-year old) patients in long-term remission (>4 years) of CS were recruited from the outpatient clinic of the department of internal medicine. Remission was defined as suppression of plasma cortisol to ≤50 nmol/L after 1 mg dexamethasone overnight [21] or, if a patient had received radiotherapy of the pituitary gland, a 24-h urinary-free cortisol excretion of <240 nmol/24 h for men or <150 nmol/24 h for women (upper levels of normal-free cortisol excretion). Clinical history was collected and a physical examination, biochemical and hormonal evaluation, dual-energy X-ray absorptiometry scanning (DXA), and non-invasive vascular function measurements were performed in all subjects. All subjects were genotyped for the presence of four GR polymorphisms; *Bcl*I, N363S, ER22/23EK, and 9*β*.

Patients with untreated hormonal deficiencies, or hormonal deficiencies that had not been treated adequately in the last 4 years according to international standards, were excluded. Furthermore, patients with active malignancy or systemic therapy for malignancy in the past, auto-inflammatory diseases, and psychiatric pathology were excluded. Hypothyroidism was defined as free thyroxine (fT4) plasma concentrations <8 pmol/l (reference range 8–22 pmol/l). Testosterone deficiency in men was defined as early morning testosterone levels <11 nmol/l (reference range 11–45 nmol/l). In women, estrogen deficiency was defined as secondary hypogonadotropic hypogonadism or a postmenopausal state without the use of chronic estrogen substitution therapy. Growth hormone (GH) deficiency was defined as a maximal GH response of <15.3 mU/l during an insulin tolerance test (ITT), or as a maximal GH response of <12.3 mU/l during an arginine/GHRH test [22]. Glucocorticoid deficiency was defined as a maximal cortisol response <550 nmol/l during an ITT [23]. All patients underwent a new 1 mg dexamethasone suppression test (or a 24-h urinary-free cortisol measurement in case of pituitary RT) before entering the study to confirm remission. All subjects were of Caucasian (Dutch) origin.

### Clinical history

Clinical history included the etiology of CS, treatment strategies (surgery, radiotherapy, medication), treatment for coexisting hormonal deficiencies, co-morbidities, and smoking habits.

### Physical examination

Anthropometric measurements including weight and height, supine systolic, and diastolic blood pressure [average of 10 measurements, every 3 min with an oscillometric sphygmomanometer (Criticon model 1846; Criticon Inc., Tampa, FL)] measured at 09:00AM.

### Biochemical evaluation

Biochemical evaluation included plasma level measurements of fasting glucose, glycated hemoglobin (HbA1c), total cholesterol, triglycerides, high-density lipoprotein (HDL), low-density lipoprotein (LDL), and insulin. Insulin sensitivity was assessed by homeostasis model assessment (HOMA) [24]. Furthermore, serum adipokines adiponectin, leptin, and resistin were measured. In addition, a number of markers of vascular health were measured in serum; soluble vascular cell adhesion molecule-1 (VCAM-1), soluble intercellular adhesion molecule-1 (ICAM-1), plasminogen activator inhibitor-1 (PAI-1), and soluble E-selectin. Increased levels of these parameters are indicators of endothelial dysfunction and worse vascular health.

### Vascular evaluation

Carotid intima media thickness (IMT) was determined using an AU5 ultrasound machine (Esaote Biomedical) with a 7.5 MHz linear array transducer. Measurement of IMT was performed off-line by the sonographer at the time of the examination, using semi-automatic edge-detection software (M’Ath^®^ Std version 2.0, Metris). IMT was defined as the mean IMT of the four measured segments of the common carotid artery: far wall left, near wall left, far wall right, and near wall right.

### Body composition

Total body DXA was performed using a Hologic QDR 4500 densitometer (Hologic, Bedford, MA). Standard procedures supplied by the manufacturer for scanning and analyses (using Hologic software version 12.1) were followed. Calibration procedures were performed every day using the appropriate phantoms provided by the manufacturer. Total body fat mass was determined and fat percentage was calculated as the total body fat mass in percent of body weight. Furthermore, trunk-, leg-, and extremities fat percentages were determined in order to discriminate between different body regions. Different regions were determined by manually placing regions of interest as defined by the manufacturers software manual. The trunk fat depot is defined as the region between two horizontal lines placed on the lower border of the head and the upper border of the iliac crest, and two vertical lines placed against the outer margins of the chest. These lines exclude the arms from the trunk. The leg fat depot is defined by two vertical lines placed against the outer margins op both legs. The pelvis is excluded from the legs by a line through the femoral neck. Leg fat percentage is depicted as the average of both legs. Extremity fat percentage is depicted as the average of both arms and both legs.

### Laboratory measurements

Serum concentrations of leptin, resistin, PAI-1, sICAM-1, and soluble E-selectin were measured by Multiplex Fluorescent Bead Immunoassays (xMAP technology, Millipore, Billerica, MA) and a Bio-plex microbead analyzer (Luminex, Austin, TX) according to the manufacturers protocol. Serum concentrations of adiponectin and sVCAM-1 were determined by enzyme-linked immunosorbent assays (R&D Systems, Minneapolis, MN). Fasting plasma glucose, HbA1c, insulin and total serum cholesterol, triglycerides, LDL-cholesterol, and HDL-cholesterol were measured by standard procedures. According to the manufacturer’s information, intra-assay precision coefficients of variation for all laboratory measurements were equal or below 5 %.

### Genetic analyses

DNA was isolated from whole blood using the Gentra Puregene isolation kit (Qiagen, Valencia, CA, USA), according to the manufacturers protocol. Genotyping for the four selected genetic variants of the glucocorticoid receptor (GR) gene (official gene name *NR3C1*) was performed as follows. The presence of the 9*β* (rs6198) and N363S (rs56149945) genetic variants was assessed by applying the predesigned TaqMan SNP assays C_8951023_10 and C_26841917_40, respectively, on a 7300 ABI Real-Time polymerase chain reaction (PCR) system (all from Life Technologies, Applied Biosystems, Foster City, CA).

For the detection of the GR ER22/23EK (rs6189/rs6190), genetic variant conventional PCR and Sanger sequencing analysis were performed with forward primer 5′-CTG-CCT-CTT-ACT-AAT-CGG-ATC-A-3′ and reverse primer 5′-AGA-GTG-AAA-CTG-CTT-TGG-ACA-G-3′. To determine the GR *Bcl*I genotype (rs41423247), DNA was amplified with forward primer 5′-AAG-CAA-TGC-AGT-GAA-CAG-TGT-AC-3′ and reverse primer 5′-AAC-AAT-TTT-GGC-CAT-CAG-TTA-TC-3′. Also these PCR products were subjected to Sanger sequencing analysis.

### Statistical analyses

Data are expressed as mean ± 95 % confidence intervals unless stated otherwise. Data distributions were analyzed using the Kolmogorov–Smirnov test, and logarithmic transformation was performed before statistical testing when appropriate. Based on data distribution, comparison of continuous variables was performed using Student’s *t* test or Mann–Whitney rank sum test. The associations between genotypes and outcome measurements were evaluated using analysis of covariance (ANCOVA) with genotypes as a factor and age, gender, and BMI as covariates. Categorical variables were analyzed using the χ^2^ test followed by Fisher’s exact test if appropriate. Hardy–Weinberg equilibrium for all polymorphisms was determined using a χ^2^ test. Significance was set at a *P* value of <0.05. Statistical analysis was performed using SPSS Software version 20.0 (SPSS, Inc., Chicago, IL).

## Results

**Fig. 1:**
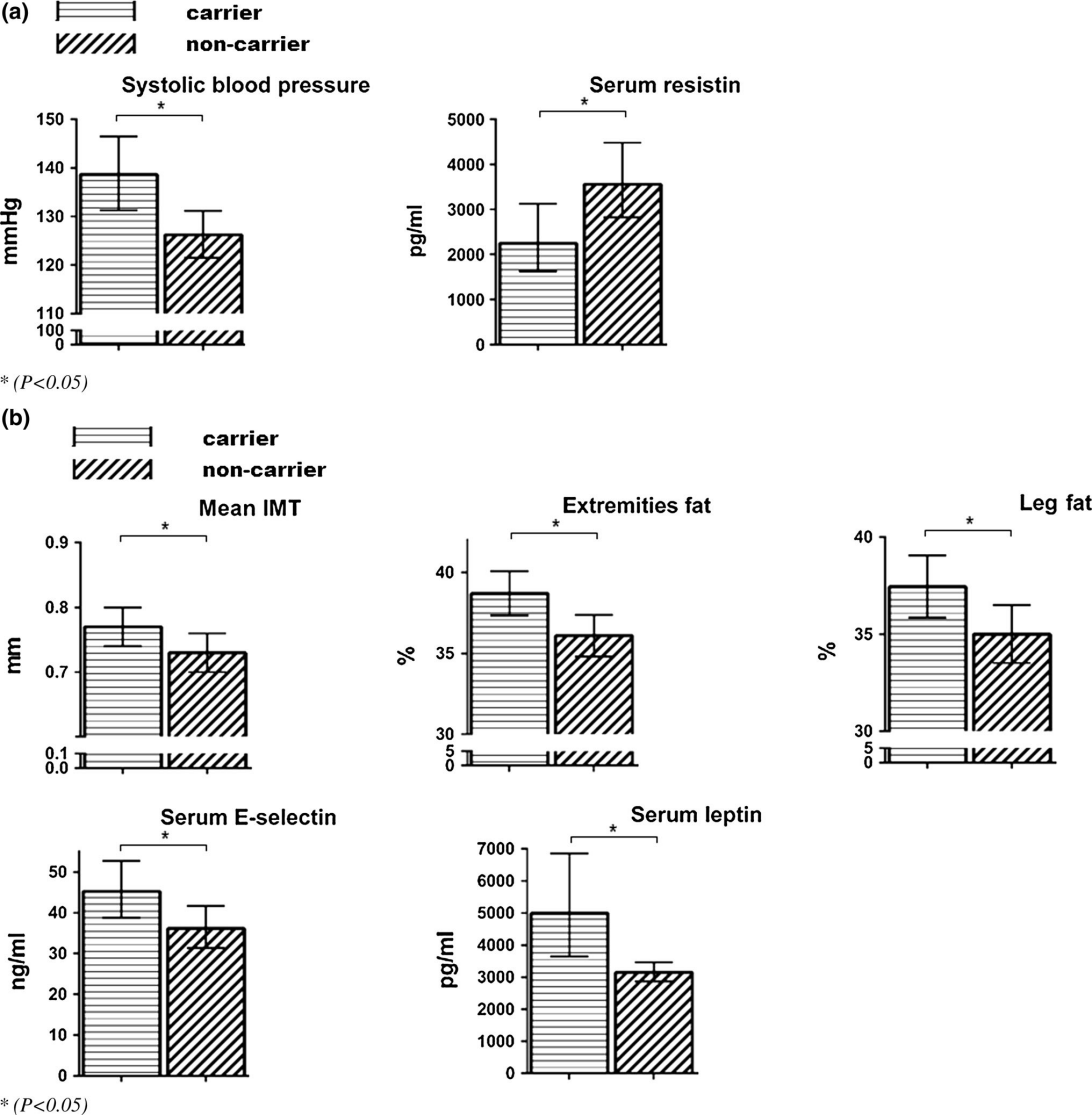
**a** Associations of the *9β* genotype and clinical outcome *P < 0.05. **b** Associations of the *Bcl*I genotype and clinical outcome *P < 0.05

Forty-eight (80 %) subjects were female. Mean (SD) age was 50.7 (12.4) years with a mean (SD) BMI of 26.9 (5.3) kg/m^2^. Mean (SD) duration of remission was 13.8 (8.5) years. Nineteen (31.7 %) subjects were treated for hypertension, 4 (6.7 %) were treated for diabetes mellitus, and 12 (20.0 %) were treated for hypercholesterolemia. Complete subject characteristics of all subjects are depicted in Table 1. Allelic frequencies of the four polymorphisms are depicted in Table 2. All polymorphisms were in Hardy–Weinberg equilibrium. Because of low allele frequencies, the N363S and ER22/23EK polymorphisms were not included in the association analysis. The minor allele of the *9β* polymorphism showed a statistically significant association with higher systolic blood pressure (*P* = 0.007) and lower resistin levels (*P* = 0.027) in carriers (Table 3; Fig. 1a). No associations were detected for the other measured parameters. The minor allele of the *Bcl*I genotype showed statistically significant associations with higher mean IMT (*P* = 0.048), higher extremities fat percentage (*P* = 0.007), higher leg fat percentage (*P* = 0.029), higher leptin level (*P* = 0.038), and higher soluble E-selectin level (*P* = 0.037) in carriers (Table 3; Fig. 1b). No associations with the other measures were detected in our CS patients (data not shown).

**Table 1:** Subject characteristics

	(*n* = 60)
Gender (*n*): male/female	12/48
Age in years: mean (SD)	50.7 (12.4)
Duration of remission: mean (SD)	13.8 (8.5)
BMI in kg/m^2^ : mean (SD)	26.9 (5.3)
Waist circumference in cm: mean (SD)	91.8 (14.7)
Smoking (*n*): yes/no	14/45
Treatment modalities: *n* (%)	
Unilateral adrenalectomy	20 (33.3)
Bilateral adrenalectomy	12 (20.0)
Pituitary surgery	38 (63.3)
Pituitary radiotherapy	13 (21.7)
Hormonal deficiencies: *n* (%)	
Glucocorticoid deficiency*	22 (36.7)
Growth hormone deficiency*	15 (25.0)
Thyroid hormone deficiency*	26 (43.3)
Testosterone deficiency*	6 (10.0)
Mineralocorticoid deficiency*	11 (18.3)
Estrogen deficiency	26 (54.2)
Cardiometabolic co-morbidities: *n* (%)	
Hypertension**	19 (31.7)
Diabetes mellitus**	4 (6.7)
Hypercholesterolemia**	12 (20.0)
Cushing type: *n* (%)	
Pituitary	40 (66.7)
Adrenal	20 (33.3)

*BMI* body mass index, *CS* Cushing’s syndrome

* Adequately substituted according to international standards

** Actively treated for this co-morbidity

**Table 2:** Genotype distributions and allele frequencies of the GR gene polymorphisms in patients cured of Cushing’s syndrome (CS)

	CS (*n* = 60)
*Bcl*I	
CC	32 (53 %)
CG	20 (33 %)
GG	8 (13 %)
Allele frequency	0.300
9*β* (A3669G)	
AA	40 (67 %)
AG	19 (32 %)
GG	1 (2 %)
Allele frequency	0.175
N363S	
AA	55 (92 %)
AG	5 (8 %)
GG	0 (0 %)
Allele frequency	0.042
ER22/23EK	
GG	55 (92 %)
GA	5 (8 %)
AA	0 (0 %)
Allele frequency	0.042

**Table 3:** Associations between glucocorticoid receptor polymorphisms and outcome measurements

	Carrier minor allele	Non-carrier minor allele	*P* value
*9β*			
Fasting glucose (mmol/l)	5.1 (4.7-5.4)	5.0 (4.7–5.2)	0.578
HbA1c (mmol/mol)	40.9 (38.4-43.6)	38.4 (36.7–40.1)	0.117
Total cholesterol (mmol/l)	5.3 (5.0–5.7)	5.2 (4.8–5.5)	0.596
Triglycerides (mmol/l)	1.4 (1.2–1.8)	1.5 (1.3–1.7)	0.850
HDL-cholesterol (mmol/l)	1.3 (1.2–1.5)	1.4 (1.3–1.4)	0.919
LDL-cholesterol (mmol/l)	3.2 (2.9–3.6)	3.1 (2.9–3.3)	0.498
Insulin (mE/l)	6.0 (4.6–7.8)	7.2 (6.0–8.7)	0.266
HOMA-IR	1.4 (1.1–1.9)	1.6 (1.3–2.0)	0.574
Mean IMT (mm)	0.73 (0.70–0.77)	0.76 (0.73–0.78)	0.254
Systolic blood pressure (mmHg)	138.7 (131.2–146.5)	126.2 (121.5–131.1)	0.007*
Total body fat (%)	32.6 (30.8–34.3)	34.3 (33.1–35.2)	0.114
Trunk fat (%)	32.3 (30.4–34.3)	33.2 (31.8–34.5)	0.471
Extremities fat (%)	35.8 (34.2–37.5)	38.1 (36.9–39.2)	0.340
Leg fat (%)	34.0 (32.1–36.1)	35.4 (34.0–36.9)	0.271
Leptin (pg/ml)	3374.5 (2289.3–4974.1)	4213.3 (3206.7–5535.9)	0.356
Resistin (pg/ml)	2248.5 (1618.1–3124.4)	3558.2 (2824.3–4482.8)	0.020*
Adiponectin (pg/ml)	706.3 (458.1–1089.0)	1087.9 (802.7–1474.4)	0.108
PAI-1 (pg/ml)	1884.7 (1377.2–2392.3)	2384.8 (2027.4–2742.2)	0.114
VCAM-1 (pg/ml)	666.5 (576.5–770.5)	674.5 609.7–746.2)	0.888
ICAM-1 (pg/ml)	253.4 (177.0–362.9)	304.3 (236.3–391.9)	0.410
E-selectin (pg/ml)	46.2 (37.9–54.5)	42.2 (36.3–48.1)	0.436
*Bcl*I			
Fasting glucose (mmol/l)	5.2 (4.9–5.5)	4.8 (4.6–5.1)	0.097
HbA1c (mmol/mol)	40.6 (38.5–42.8)	38.1 (36.2–40.0)	0.089
Total cholesterol (mmol/l)	5.2 (4.9–5.6)	5.2 (4.9–5.5)	0.837
Triglycerides (mmol/l)	1.6 (1.4–1.9)	1.3 (1.1–1.6)	0.198
HDL-cholesterol (mmol/l)	1.4 (1.2–1.5)	1.4 (1.3–1.4)	0.969
LDL-cholesterol (mmol/l)	3.1 (2.9–3.4)	3.2 (2.9–3.4)	0.879
Insulin (mE/l)	7.4 (5.9–9.2)	6.3 (5.1–7.8)	0.313
HOMA-IR	1.8 (1.4–2.3)	1.4 (1.1–1.7)	0.125
Mean IMT (mm)	0.77 (0.74–0.80)	0.73 (0.70–0.76)	0.048*
Systolic blood pressure (mmHg)	126.0 (120.2–132.0)	134.0 (128.3–14.−1)	0.060
Total body fat (%)	34.5 (33.0–36.0)	33.1 (31.7–34.4)	0.167
Trunk fat (%)	33.3 (31.7–34.9)	32.5 (31.0–34.1)	0.488
Extremities fat (%)	38.7 (37.3–40.1)	36.1 (34.8–37.4)	0.007*
Leg fat (%)	37.5 (35.8–39.1)	35.0 (33.5–36.5)	0.029*
Leptin (pg/ml)	4999.0 (3644.6–6856.8)	3155.8 (2869.8–3470.3)	0.038*
Resistin (pg/ml)	3630.0 (2738.1–4812.6)	2625.4 (2016.3–3418.7)	0.099
Adiponectin (pg/ml)	1166.8 (811.6–1677.4)	781.3 (556.1–1097.7)	0.112
PAI-1 (pg/ml)	2301.6 (1866.2–2737.1)	2145.0 (1737.8–2552.2)	0.602
VCAM-1 (pg/ml)	726.3 (645.5–817.3)	627.7 (562.3–700.6)	0.075
ICAM-1 (pg/ml)	282.3 (208.5–382.2)	289.5 (218.1–384.1)	0.904
E-selectin (ng/ml)	45.2 (38.8–52.7)	36.1 (31.3–41.7)	0.037*

Data are expressed as mean and 95 % confidence intervals

All data are adjusted for age, gender, and BMI

*IMT* intima media thickness, *HbA1c* glycated hemoglobin, *HDL* high-density lipoprotein, *LDL* low-density lipoprotein, *HOMA-IR* homeostatic model assessment-insulin resistance, *PAI* plasminogen activator inhibitor, *VCAM* vascular cell adhesion molecule, *ICAM* intracellular adhesion molecule

* P ANCOVA was used to assess the statistical differences between genotype groups, *P* < 0.05 (bold)

## Discussion

This study investigated the prevalence of GR polymorphisms in patients in long-term remission of CS and the associations of these polymorphisms with metabolic, vascular, and body compositional characteristics. In this study, we found that carriers of the 9*β* polymorphism have a higher systolic blood pressure and lower resistin levels and that the GC sensitizing *Bcl*I polymorphism is associated with a number of metabolic and vascular adverse effects: higher fat percentages of extremities and legs, higher serum leptin, E-selectin levels, and higher intima media thickness in carriers versus non-carriers.

It has been shown that in patients in long-term remission of CS, cardiovascular and metabolic risk remain increased [3, 5]. In the general population, it has been demonstrated that cardiovascular and metabolic risk and body composition are affected by lifelong overactivation or relative inactivation of GC signaling due to GR polymorphisms [25]. Our observation that altered glucocorticoid sensitivity due to GR polymorphisms modulates cardiometabolic risk factors in cured CS patients is in line with these well-known findings in the general population.

Remitted CS patients carrying the minor allele of the 9β polymorphism had a higher systolic blood pressure, which is concordant with previous findings of an increase in carotid atherosclerosis, and a higher incidence of coronary heart disease in carriers of this polymorphism [17]. Systolic hypertension is normally especially seen in the elderly population due to increased arterial stiffening. Arterial stiffness is central to the pathogenesis of isolated systolic hypertension and directly impacts left ventricular afterload, pressure pulsatility in the arterial tree, and its penetration into the microvasculature of target organs such as the brain and kidney. This means that systolic hypertension in carriers of the polymorphism may lead to increased aging of the vascular tree, but further studies are needed to elucidate whether the polymorphism increases cardiovascular risk. Furthermore, an association was found between the 9*β* polymorphism and resistin levels, with carriers of the minor allele of the 9*β* polymorphism having lower resistin levels. It can be hypothesized that this is caused by the fact that subjects with a relative decrease in glucocorticoid sensitivity have less central adiposity. Central adiposity causes local ischemia of the adipose tissue which leads to infiltration of M1-macrophages, which are the main producers of resistin. This hypothesis is, however, speculative and not supported by the data, since no association between truncal fat and the 9β polymorphism was observed.

The minor allele of the *Bcl*I polymorphism was associated with an increased fat percentage of the extremities and legs in the remitted CS patients. These body areas only contain subcutaneous adipose tissue. As leptin is preferentially produced in subcutaneous adipose tissue (SAT), carriers also had higher leptin levels [26]. High leptin levels have been widely recognized as an independent cardiovascular risk factor associated with insulin resistance. It also has a pathogenic role in atherothrombosis and endothelial dysfunction. Furthermore, a higher level of E-selectin was found in carriers of the minor allele of the *Bcl*I polymorphism. This may reflect increased endothelial activation and progressing atherosclerosis in the *Bcl*I carriers. This is supported by our observation that the IMT is higher in *Bcl*I carriers.

In women with CS in remission, the *Bcl*I polymorphism was previously shown to be associated with altered GC sensitivity as the polymorphism was associated with reduced total and femoral neck bone mineral density [27]. Furthermore, in patients in remission of CS, the *Bcl*I polymorphism was also independently associated with increased fatigue and worse performance on cognitive testing [20]. The findings in literature combined with the findings in the current study suggest that hypercortisolism in patients carrying the *Bcl*I polymorphism may have more pronounced effects on patient wellbeing in the long term.

This study has some limitations. Because of the low incidence of CS, only a relatively small cohort could be studied. This could influence genetic associations and that is why only polymorphisms with a relatively high frequency were included in the association analysis. In genetic association studies, inhomogeneity of study population with regard to ethnicity, gender, age, and environmental factors is a frequent limiting factor. This study included Caucasians only and associations were corrected for gender and age. Duration of hypercortisolism likely represents another factor in long-term cardiometabolic effects in CS patients, which, however, is rather difficult to estimate since diagnosis of CS is generally delayed. Furthermore, it was decided not to correct for multiple testing because of the hypothesis-driven nature of this study. Indeed, from a biological perspective our findings are in line with the expected corticosteroid effects, suggesting that SNPs of the GR gene leading to either hypo- or hypersensitivity to GC, indeed modulate long-term cardiometabolic outcome after treatment of CS. However, because of the limited sample size and, hence, limited statistical power, we cannot exclude that some of the significant differences between carriers and non-carriers may be a finding by chance, i.e., a type-1 error.

In conclusion, this study is one of the first to suggest that glucocorticoid receptor polymorphisms modulate cardiometabolic risk factors in patients in long-term remission of CS [27]. In the context of personalized healthcare, this may implicate that, after treatment of CS, carriers of these polymorphisms are candidates for a more stringent follow-up regarding cardiovascular and metabolic health as our findings suggest that GR polymorphisms may play a role in susceptibility to cardiovascular disease in CS patients. Furthermore, patients who treated with glucocorticoids for other diseases and even healthy subjects, carrying these polymorphisms, are candidates for a more stringent follow-up regarding cardiovascular and metabolic health, and the development of metabolic syndrome as was recently debated in literature [28]. However, the results of this study need to be interpreted with caution, and further research into these findings in larger CS populations is needed to replicate these findings. In addition, future studies should delineate to what extent the observed associations also apply to prolonged episodes of exposure to physiologically elevated cortisol levels (e.g., severe stress) or exogenous glucocorticoids.
